# A Coupled Field Multiphysics Modeling Approach to Investigate RF MEMS Switch Failure Modes under Various Operational Conditions

**DOI:** 10.3390/s91007988

**Published:** 2009-10-12

**Authors:** Khaled Sadek, Jonathan Lueke, Walied Moussa

**Affiliations:** Department of Mechanical Engineering, University of Alberta / University of Alberta, Edmonton, AB, T6G 2G8, Canada; E-Mails: kmahmoud@ualberta.ca (K.S.); lueke@ualberta.ca (J.L.)

**Keywords:** RF MEMS switch, substructuring, skin effect, buckling, residual stresses

## Abstract

In this paper, the reliability of capacitive shunt RF MEMS switches have been investigated using three dimensional (3D) coupled multiphysics finite element (FE) analysis. The coupled field analysis involved three consecutive multiphysics interactions. The first interaction is characterized as a two-way sequential electromagnetic (EM)-thermal field coupling. The second interaction represented a one-way sequential thermal-structural field coupling. The third interaction portrayed a two-way sequential structural-electrostatic field coupling. An automated substructuring algorithm was utilized to reduce the computational cost of the complicated coupled multiphysics FE analysis. The results of the substructured FE model with coupled field analysis is shown to be in good agreement with the outcome of previously published experimental and numerical studies. The current numerical results indicate that the pull-in voltage and the buckling temperature of the RF switch are functions of the microfabrication residual stress state, the switch operational frequency and the surrounding packaging temperature. Furthermore, the current results point out that by introducing proper mechanical approaches such as corrugated switches and through-holes in the switch membrane, it is possible to achieve reliable pull-in voltages, at various operating temperatures. The performed analysis also shows that by controlling the mean and gradient residual stresses, generated during microfabrication, in conjunction with the proposed mechanical approaches, the power handling capability of RF MEMS switches can be increased, at a wide range of operational frequencies. These design features of RF MEMS switches are of particular importance in applications where a high RF power (frequencies above 10 GHz) and large temperature variations are expected, such as in satellites and airplane condition monitoring.

## 1. Introduction

Radio Frequency (RF) Micro-Electro-Mechanical Systems (MEMS) capacitive shunt switches have had an enormous impact on the applications of wireless communication systems and satellite technology. These switches raise the performance of communication systems to a level significantly above their solid-state counterparts such as FETs and PIN diodes. The prime reasons that these RF MEMS switches are currently under immense consideration includes, their wider capacity frequency range (from RF to mm-wave signal), lower power consumption, lower insertion loss, and lack of inter-modulation distortion [1-4]. The reliability of these switches are reported to sustain up to 0.1–40 billion cycles, while only handling power with maximum capacity of 0.5 W [4-7]. The major failure modes of RF MEMS switches are shown to occur due to membrane stiction, buckling, and creep [5].

The operation of RF MEMS switches requires an electrostatic force in order to actuate from the up, or “off” position, into the down, or “on” position. The voltage required to actuate the RF MEMS switch in this manner is referred to as the pull-in voltage. This voltage is dictated by the geometry and stiffness of the RF MEMS switch. A higher pull-in voltage will decrease the overall lifespan of the RF MEMS switch, as shown by [1].

As in all micromachined MEMS devices, residual stresses develop in the manufacturing process. The residual stresses can be separated into two specific stresses, thermal and intrinsic stresses [8-10]. Thermal stresses are generated through differences in thermal expansion coefficients in adjacent materials in the manufacturing process. The generation of intrinsic stresses is far more complicated. Generally, intrinsic stresses involve any stress generated from excess vacancies, crystal dislocations, grain boundary interactions, or phase transformations [8,10]. Overall, the residual stresses generated in RF MEMS switches ultimately affect the general state of stress, and therefore, failure mechanisms of the MEMS device. While loaded with high frequency alternating current, the RF MEMS switch undergoes an electron crowding phenomena, otherwise known as the skin effect [5]. Due to self-inductance, the electrons flowing through the switch will be crowded on the edges of the conductor, creating a localized area of high temperature near the edges of the RF switch. Therefore, severe compressive thermal stresses develop due to this localized heating, causing the switch to buckle upwards. Depending upon the magnitude and direction of the residual stresses, this localized heating could potentially cause the RF MEMS switch to fail under severe buckling with sufficient load.

The geometry of the RF MEMS switch greatly affects the power handling and frequency loads that each switch can experience. In addition, the actuation voltage of RF MEMS switches is affected by the geometry of these switches; for example, a mechanically stiffer switch will require a larger pull-in voltage. Mechanical approaches such as the addition of corrugations and holes can be introduced to control the membrane stiffness of the RF MEMS switch, in order to attain a specific design pull-in voltage [4].

In this paper, a multi-domain 3D FE model is developed to investigate the multiphysics interactions within capacitive shunt RF MEMS switches. This coupled field model studies the electromagnetic, thermal, structural and electrostatic physics of RF MEMS switches, in response to variations within geometry, residual stresses (mean and gradient) and operational current frequency. The obtained results are then utilized to predict the onset of stiction and buckling with RF MEMS switches and its effect on these switches long term reliability.

## 2. Coupled Field FE Analysis of RF MEMS Switch

**Figure 1.**
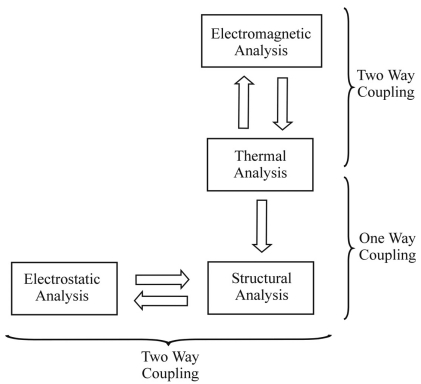
Flow chart for coupled field analysis procedure for RF MEMS switch.

A flowchart illustrating the analysis procedure of the RF MEMS switch coupled field FE model, is shown in Figure 1. The full model was solved numerically using ANSYS 10.1 indirect multiphysics solver [11].

**Figure 2.**
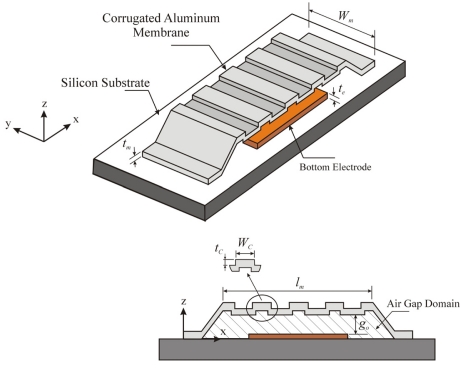
Corrugated RF MEMS switch.

For the current study, the RF switch model consists of corrugated aluminum membrane, silicon substrate and bottom electrode, as shown in Figure 2.

First, the distribution of the current density (*J*) is calculated at a frequency (ω) range of 0.1–100 GHz by conducting a full wave harmonic electromagnetic (EM) analysis. The heat generated in the switch membrane is calculated from *J*, and then used to calculate the temperature distribution (*T*) through a steady state thermal analysis. The EM-thermal analysis was performed as a sequentially coupled field analysis involving two-way multiphysics coupling (i.e., iterations are performed between both fields). Second, a coupled thermal-structural analysis is performed to calculate the in-plane thermally induced stresses and resulting deformation in the switch geometry. The coupled thermal-structural analysis involves one-way multiphysics coupling between both fields. Finally, the pull-in voltage (*V_PI_*) for the new switch configuration, was calculated using a two-way coupled structural-electrostatic FE analysis.

### 2.1. EM-Thermal Fields Coupling

In the full wave EM field, a current (*I*) load that varies harmonically with time, is applied at the edge (x=0) of the switch membrane (Equation 1). The electric field distribution is determined by solving the governing Maxwell's equations with two known BCs (Equation 2):

(1) $$\begin{array}{cc} I=I_{o}\sin(\omega t), & x=0 \end{array}$$

(2) $$\begin{array}{c} \nabla E=\frac{\rho_{m}(T)}{{ɛ}_{o}},\nabla_{x}E=-\frac{\partial B}{\partial t},\nabla B=0,\text{and}\nabla_{x}B=\mu_{o}J+{ɛ}_{o}\mu_{o}\frac{\partial E}{\partial t}\\ \text{BCs},E={0|}_{x=l_{m}},{\vec{B}.\vec{s}=0|}_{x=l_{m}} \end{array}$$

where, *I_o_* is the current amplitude; ∇, is the divergence operator; *E*, is the electrical field strength; *ρ_m_(T)*, is the temperature dependant electrical resistivity of the switch membrane material; *ε_o_*, is the permittivity of free space; ∇*_x_*, is the curl operator; *B*, is the magnetic field strength; and *μ_o_* is the permeability of free space.

After solving for *E*, the current density is calculated by Equation 3, as follows:

(3) $$J=\frac{E}{\rho_{m}(T)}$$

Once the current density is calculated from the EM field, the model is switched to the thermal field. The heat generation (*H*) throughout the beam is calculated by Equation 4:

(4) $$H={\left\|J\right\|}^{2}\rho_{m}(T)$$

For the frequencies considered in this study (*ω* ≥ 0.1 GHz), the time constant for the thermal response is much longer than the period of variation of the input power. Therefore, the temperature distribution throughout the switch membrane is calculated using the steady state heat equation given by [5]:

(5) $$\nabla (k_{m}\nabla T)=-{\left\|J\right\|}^{2}\rho_{m}(T)$$

where, *k_m_* is the thermal conductivity of the switch membrane material. The convection and radiation heat transfer mechanisms were negligible compared to the heat transfer by conduction. Therefore, only conductive heat transfer was considered in the thermal analysis. Same assumption has been validated in a previous study reported by Jensen *et al.* [5].

For the thermal model, the boundary conditions (BCs) assume that the edges of the switch membrane (*x* = 0, *x* = *l_m_*) are kept at room, or in other words the reference temperature (*T_r_*). Adiabatic BCs are assumed for the other four sides of the membrane (*z* = *g_o_, z* = *g_o_* + *t_m_, y* = 0, and *y* = *W_m_*).

Once the temperature distribution is found, a new value for the electrical resistivity is calculated and the analysis is switched back to the EM field to update the current density distribution. Iterations between the EM and thermal fields are executed in sequence until a convergence for the value of *T* is achieved. The current applied to the EM field is ramped up and the whole procedure is repeated until the value of the maximum current is reached.

### 2.2. Thermal-Structural Fields Coupling

The temperature distributions calculated from the coupled EM-Thermal FE analysis are applied to the structural field model as body loads. The coupling between the thermal and structural fields was conducted in a one-way sequential interaction manner. The model was completely switched to the structural field after the thermal analysis has been fully conducted. No iterations were performed between the two fields. Due to the mismatch of the coefficient of thermal expansion between the switch membrane and the substrate, thermal stresses and a new switch deformation state results from the applied *T*. The thermally induced axial strain (*ε_th,x_*) and stress (*σ_th,x_*) are calculated at a given position (x, z) by [5]:

(6) $${ɛ}_{th,x}(x,z)=-\Delta \alpha \int_{T_{r}}^{T(x,z)}dT,\sigma_{th,x}(x,z)=\bar{E_{m}}({ɛ}_{th,x}(x,z))$$

where, Δα is the difference in thermal coefficient expansion between the membrane and the substrate; *E̅_m_* is the effective Young's modulus of the switch membrane, given by *E̅_m_* = *E_m_*/(1-*v_m_^2^*) for *W_m_* > 5*t_m_* [12]; *E_m_* and *v_m_*, refer to the membrane material Young's modulus and Poisson's ratio, respectively.

Once the thermal induced stress distribution is evaluated, the new switch geometry is found by calculating the membrane deflection (*D_m_*) from Karaman equation, which can be expressed in 1-D form as follows [13]:

(7) $$\begin{array}{c} \frac{d^{4}D_{m}}{dx^{4}}=\frac{1}{D}\left[\frac{E_{m}t_{m}}{2}{\left(\frac{dD_{m}}{dx}\right)}^{2}\left(\frac{d^{2}D_{m}}{dx^{2}}\right)+\sigma_{th,x}(x,z)(1-\nu_{m})t_{m}\left(\frac{d^{2}D_{m}}{dx^{2}}\right)\right]\\ D_{m}={0|}_{x=0,L_{m}}\text{and}\frac{dD_{m}}{dx}={0|}_{x=0,L_{m}} \end{array},$$

where, *D* is the flexural rigidity of the membrane, given by:

(8) $$D=\frac{E_{m}t_{m}^{3}}{12(1-\nu_{m}^{2})}$$

The first term in right hand side of Equation 7 represents strain stiffening, and is considered only for large membrane deflections [12,14].

### 2.3. Structural-Electrostatic Fields Coupling

**Figure 3.**
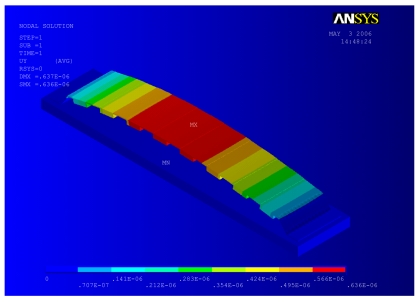
Distribution of displacement vertical component along x-coordinate predicted by coupled EM-thermal analysis followed by a coupled thermal-structural analysis for corrugated aluminum RF MEMS switch membrane (*l_m_* = 91*μm, W_m_* = 20*μm, t_m_* = 0.4*μm, t_c_* = 4*t_m_, N_c_* = 13, *ω* = 20 GHz, *P* = 0.75 W).

The non-uniform thermal induced stresses calculated from thermal-structural coupled field analysis results in a new configuration for the switch geometry, as shown in Figure 3. The new geometry is used as a starting point for the structural-electrostatic analysis. Initially, mean and gradient residual stresses measured during microfabrication [8], were applied to the switch to account for the final geometry and state of stress before proceeding to the coupled structural-electrostatic analysis.

In the electrostatic field, the essential BCs are the applied ramped voltages at the bottom and top conductors. Since the width (*W_m_*) of the modeled switch membrane is greater than the initial gap length (*g_o_*), the fringing field effects were neglected [5,12,15,16]. The voltage distribution was calculated by solving Laplace equation with two known BCs [13]:

(9) $$\begin{array}{rr} \nabla^{2}V & =\frac{\partial^{2}V}{\partial x^{2}}+\frac{\partial^{2}V}{\partial y^{2}}+\frac{\partial^{2}V}{\partial z^{2}}=0,\\ V & ={V_{0}|}_{z=g_{0}+t_{e}}\text{and}\;V={0|}_{z=t_{e}} \end{array}$$

where *V* refers to the voltage distribution and *V_o_* is the applied ramped voltage at the bottom of the aluminum switch membrane.

The electrostatic force distribution was then calculated, and the model is switched to the structural field. A new deformation state was obtained from the structural analysis and the model was switched back to the electrostatic field. Morphing BCs were applied at the side and top boundaries of the underlying medium to account for the new deformation state of the switch [11]. The electrostatic field mesh was updated and a new value for the electrostatic force distribution was calculated. Iterations between the structural and electrostatic fields are executed in sequence until a convergence was reached. The applied voltage is ramped up and the process is repeated for the new voltage value. The pull-in voltage (*V_PI_*) was finally identified when a convergent solution was not achievable [12,13].

## 3. Automated-Substructuring Algorithm

An automated substructuring algorithm was developed, in the current study, to reduce the computational cost of the coupled multi-field RF switch model. The same algorithm was utilized, in a previous study reported by the authors, to solve the coupled electro-thermal FE problem of MEMS gas sensors [17]. In this study, the reported computational savings were in the range of 59–77 percent. Full details of the automated substructuring algorithm are beyond the scope of this paper, and are briefly explained in this Section. In the developed algorithm, nonlinear criteria are set in each physics environment to identify the nonlinear regions in the FE model. These regions are isolated, and spectral techniques are used to automatically identify the locations of the linear regions in the FE mesh. The linear regions are then condensed in to a number of substructures, which are used later in the FE analysis. The generated substructures aspect ratio (AR) should be kept within a certain limit (1–10), in order to achieve accurate simulation results [18]. Therefore, the AR of the generated substructures is controlled within the specified limits using a modified version of the recursive spectral bisection (RSB) algorithm [19]. The stiffness of the generated substructures is fixed during the solution phase of the coupled field model. Therefore, only the stiffness of the nonlinear regions is updated during FE iterations, which considerably reduces the computational cost of the coupled FE analysis. Using the developed substructuring algorithm in the current study, computational savings ranging from 49–56 percent were achieved.

## 4. Validation Examples

To assess the accuracy of the substructured multi-field coupled model, three examples were performed in this study. The first two examples are designed to validate the substructured EM-thermal model. The third example is utilized to validate the substructured structural-electrostatic model.

### 4.1. Example 1

**Figure 4.**
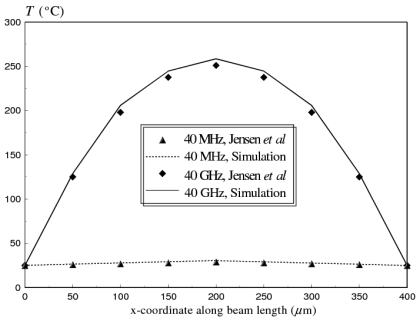
Substructured EM-thermal model predictions versus numerical model results reported by Jensen *et al.* [5] of the temperature distribution versus coordinate along beam length for fixed-fixed gold beam at frequencies of 40 MHz and 40 GHz (*l_m_* = 400 *μm, W_m_* = 50 *μm, t_m_* = 2 *μm*).

In this example, the temperature distribution along the length of a fixed-fixed gold beam (*l_m_* = 400 *μm, W_m_* = 50 *μm, t_m_* = 2 *μm*), with an input power of 1W is calculated. The beam was analyzed at two operational frequencies, 40 MHz and 40 GHz. At low frequencies, the current is spread evenly through the beam. For higher frequencies, self inductance of the conductor causes electrons crowding at the outside edges of the beam, a phenomenon commonly known as the skin effect [5]. This phenomenon increases the heat dissipation to the beam; therefore higher temperatures are generated in the beam with higher operational frequencies. Results from the substructured coupled EM-thermal model highlight this effect, and are compared with numerical model data reported by Jensen *et al.* [5], in Figure 4.

In their study, Jensen *et al.* [5] modeled the switch in the upstate position using an iterative coupled field analysis. In their approach, the current density on the cross section of the beam is calculated using the finite element-boundary integral method. The calculated current density is then used to solve the steady state heat equation using 2-D finite element simulation. The two physics were calculated in an iterative manner until the solution converges to a constant temperature profile within 1% between iterations [5]. For more details, interested readers can refer to the study reported by Jensen *et al.* [5]. A good agreement between the two models is evident, as shown in Figure 4.

### 4.2. Example 2

**Figure 5.**
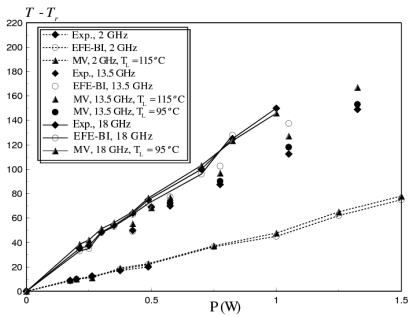
Substructured EM-thermal model predictions versus numerical model results and experimental data reported by Wang *et al.* [20] of spatially average temperature rise versus input power for fixed-fixed gold beam at frequencies of 2 GHz, 13.5 GHz and 18 GHz (*l_m_* = 400 *μm, W_m_* = 20 *μm, t_m_* = 2 *μm*).

In this example, the spatial average temperature rise versus input power in a fixed-fixed gold beam (*l_m_* = 400 *μm, W_m_* = 20 *μm, t_m_* = 2 *μm*), is calculated. The beam was analyzed at operational frequencies of 2, 13.5 and 18 GHz. The results from the substructured EM-thermal model were compared with numerical and experimental data reported by Wang *et al.* [20]. The comparison is shown in Figure 5, where, EFE-BI refers to the finite element boundary integral method used to analyze the same problem by Wang *et al.* in 2006.

The results from the Major Vector algorithm were calculated initially based on a linearity temperature of 115 °C. For an operational frequency of 13.5 GHz, the results start to diverge from the experimental measurements at an input power higher than 0.5 W. Therefore, the linearity temperature criteria were reduced to 95 °C for input power higher than 0.5 W, and the same criteria was used to calculate the spatial average temperature rise for frequencies above 13.5 GHz. As shown in Figure 5, the reduction of *T_L_*, yields better solution accuracy, but this was on the expense of higher computational costs.

### 4.3. Example 3

In this example, results from the substructured structural–electrostatic model are compared with the closed-form analytical expression for the pull-in voltage given by [12]:

(10) $$V_{PI}=\sqrt{\frac{8K_{\text{eff}}g_{o}^{3}}{27{ɛ}_{o}A_{\text{eff}}}}$$

where *K_eff_* is the effective beam stiffness, and is given by Equation 11 for a uniform electrode [12]; *ε_o_* is the permittivity of free space; *A_eff_* is the effective area, given by Equation 12 [12,15,16], which mainly accounts for the fringing field effects and the charge redistribution effects:

(11) $$K_{\text{eff}}=\left[\frac{32\bar{E_{m}}W_{m}t_{m}^{3}}{l_{m}^{3}}+\frac{8.32N}{l_{m}}\right],N=(\bar{\sigma_{\text{res}}}+\sigma_{NL})W_{m}t_{m}$$

(12) $$\begin{array}{c} A_{\text{eff}}=W_{m,\text{eff}}t_{m}\\ W_{m,\text{eff}}(\beta )=W_{m}\left(1+0.65\frac{(1-\beta )g_{o}}{W_{m}}\right),\beta =\frac{z_{\max}}{g_{o}} \end{array}$$

where, *σ̅_res_* = *σ_res_*(1-ν*_m_*) is the residual film stress; *σ_NL_* = *π^2^E_m_z_max_^2^*/4*l_m_^2^* is an estimate of the induced axial stress due to the nonlinear stiffening; *z_max_* is the center deflection of the beam at a given applied voltage.

**Figure 6.**
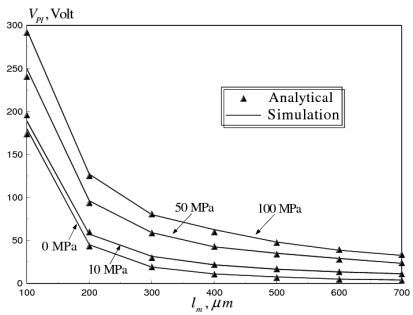
Substructured structural-electrostatic model predictions versus analytical results reported by Pamidighantam *et al.* [12] of pull-in-voltage versus beam length for fixed-fixed gold beam at different values of microfabrication residual stresses (*W_m_* = 50 *μm, t_m_* = 1 *μm, g_o_* = 2.5 *μm, β_PI_* = 0.4, *E_m_* = 77 GPa).

The analytical and the FE simulation results, for the pull-in voltage versus beam length, for a fixed-fixed gold beam, at different microfabrication residual stresses are shown in Figure 6. The comparison shows a good agreement between the substructured structural-electrostatic model and the analytical solution.

## 5. Results and Discussion

**Figure 7.**
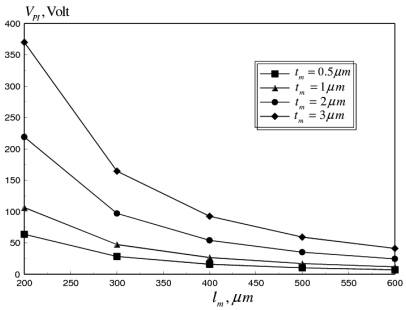
Pull-in-voltage versus membrane length at different membrane thickness of flat aluminum RF MEMS switch (*σ_M_* = *σ_G_* = 0 MPa, *W_m_* = 20 *μm, T(x)* = *T_r_*).

**Figure 8.**
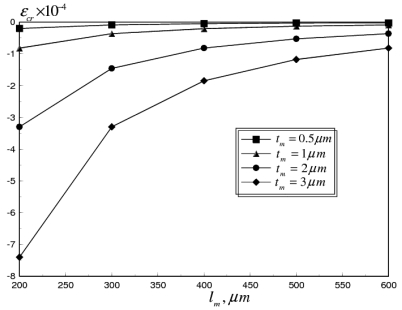
Critical buckling strain versus membrane length at different membrane thickness of flat aluminum RF MEMS switch.

Given the good accuracy exhibited by the coupled multi-field FE model, the reliability of aluminum RF MEMS switches is examined in this section. Two candidate mechanisms are studied in this paper, mainly stiction problem at higher pull-in voltages and failure due to buckling. First, the effect of the operational frequency in the range of 0.1–100 GHz is studied, for flat switch membranes. Next, the effect of mean and gradient residual stresses, generated during different stages of microfabrication is evaluated. Finally, corrugated switch membranes and through holes in the switch membrane are presented, as alternative mechanical solutions for the RF switch reliability problem. The variation of the pull-in voltage (*V_PI_*) and critical buckling strain (*ε_cr_*) with switch length (*l_m_*) at different membrane thicknesses (*t_m_* = 0.5 – 3 *μm*), are shown in Figures 7 and 8, respectively.

The pull-in voltage was calculated for a flat aluminum membrane, at room temperature, in the absence of residual stresses. It can be seen that *V_PI_* decreases with the increase in *l_m_* due to the reduction in the bending stiffness of the switch membrane [12]. The same effect can also be noticed for *ε_cr_*, which varies proportionally with (*t_m_*/*l_m_*)^2^.

### 5.1. Effects of Operational Frequencies

**Figure 9.**
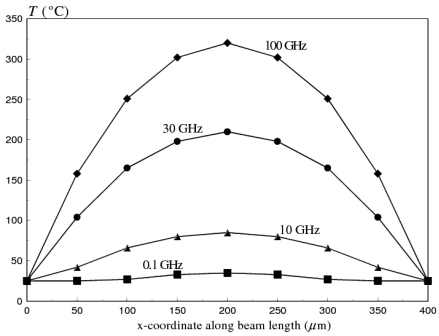
Temperature distribution along membrane length of flat aluminum RF MEMS switch at an operating frequency range of 0.1-100 GHz (*l_m_* = 400 *μm, W_m_* = 20 *μm, t_m_* = 3 *μm, P* = 0.75 W)

The steady state temperature distribution along switch membrane length at an input power of 0.75 W, is plotted in Figure 9, as a function of operational frequency.

**Figure 10.**
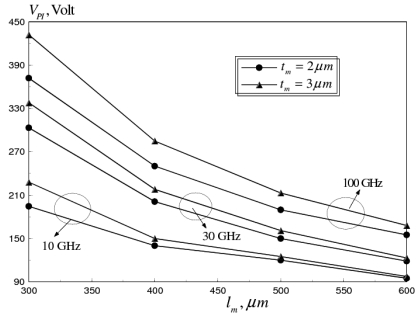
Pull-in voltage versus membrane length of flat aluminum RF MEMS switch at operating frequencies of 10, 30 and 100 GHz (*W_m_* = 20 *μm, t_m_* = 2 and 3 *μm*).

The maximum temperature occurs at the mid span of the beam. However, the maximum steady state temperature at 0.1 GHz (29.5 °C) is considerably lower than at 100 GHz (320 °C), due to the skin effect. The temperature rise induces compressive thermal stresses in the switch membrane, which increases the initial gap between the membrane and the bottom electrode (see, Figure 3). As a consequence, a coupled structural-electrostatic analysis of the switch membrane shows that *V_PI_* increases dramatically with the increase in *ω*, because of the change in the initial deformation state of the switch, as shown in Figure 10. Moreover, thermally induced compressive stresses cause the switch to buckle at *ω* ≥ 10 GHz.

### 5.2. Effects of Residual Stresses

**Figure 11.**
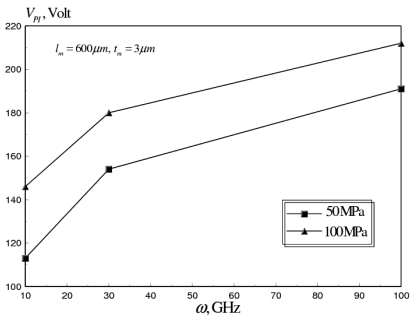
Effect of microfabrication mean residual stresses on *V_PI_* of flat aluminum RF MEMS switch at operating frequency range of 10-100 GHz (*l_m_* = 600 *μm, W_m_* = 20 *μm, t_m_* = 3 *μm*).

Residual stresses are inherently generated during various stages of microfabrication. A general uniaxial residual stress field can be approximated by [21,22].

(13) $$\begin{array}{l} \sigma_{\text{res}}(z)\approx \sigma_{M}+\sigma_{Gr}(z),\\ g_{o}\leq z\leq g_{o}+t_{m} \end{array}$$

where, *σ_M_, σ_Gr_(z)* refer to the mean and gradient (intrinsic) residual stresses generated in the thin film during microfabrication, respectively. Typical values reported in the literature indicate experimental measurements in the range of 0 to over 150 MPa for *σ_M_* and 5–30 MPa for *σ_Gr_(z)* [8]. Uniaxial tensile mean residual stresses (50–100 MPa), were applied to the flat aluminum RF switch to examine its effect on *V_PI_* and average axial strain (*ε*). The pull-in voltage-frequency curves predicted by the multi-field coupled model are shown in Figure 11, at different values of *σ_M_* for flat RF switch membrane with dimensions (*l_m_* = 600 *μm, W_m_* = 20 *μm, t_m_* = 3 *μm*).

**Figure 12.**
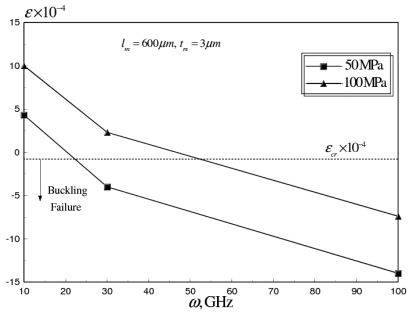
Effect of microfabrication mean residual stresses on *ε* of flat aluminum RF MEMS switch at operating frequency range of 10-100 GHz (*l_m_* = 600 *μm, W_m_* = 20 *μm, t_m_* = 3 *μm*).

It can be seen that the applied residual stresses increase the pull-in voltage by 20–40 percent. This increase can be explained by the increased stiffening of the beam due to the increase in the value of the applied residual stress. However, as shown in Figure 12, the tensile residual stresses counteract the compressive thermal stresses induced at higher operational frequencies.

It can be seen that *ε* can be shifted above the value of the critical buckling strain, which increases the power handling capability of the switch, to higher operational frequencies. Therefore, the microfabrication tensile residual stresses can be utilized to eliminate the buckling failure. Conversely the stiction problem, due to charge build-up in the dielectric layer associated with higher actuation voltages, can still cause switch failure at a limited number of operational cycles.

### 5.3. Mechanical Approaches

As discussed in subsection 5.2, a proper control of tensile residual stresses generated during microfabrication, can be utilized to increase the power handling capabilities of RF MEMS switches. However, the higher actuation voltages exhibited; because of membrane stiffening is still a major problem. Therefore, to achieve reliable switch designs, much lower actuation voltages should be achieved. Three strategies have been proposed in the literature to achieve this goal. The first one consists in modifying the out-of-plane geometry of the switch membrane [13]. The second strategy consists in the use of corrugated switch membranes [13]. The third strategy consists in the addition of holes to reduce the membrane effective Young's modulus [22]. The second and third strategies are more achievable in microfabrication [13]; hence, the combination of both strategies in RF MEMS switch design is examined in this subsection, for an RF MEMS switch with dimensions (*l_m_* = 600 *μm, W_m_* = 20 *μm, t_m_* = 3 *μm*).

**Figure 13.**
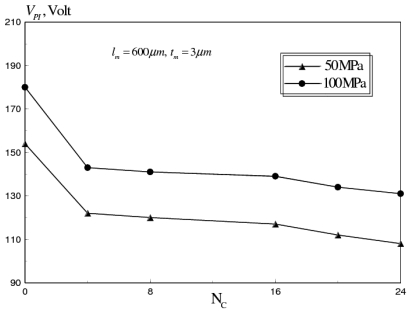
Effect of number of corrugations on *V_PI_* of corrugated aluminum RF MEMS switch at *σ_M_* range of 50–100 MPa (*l_m_* = 600 *μm, W_m_* = 20 *μm, t_m_* = 3 *μm, t_C_/t_m_* = 0.16, *ω* = 30 GHz).

First, the effect of number of corrugations (*N_C_*) and corrugation thickness (*t_C_*), expressed by corrugation thickness ratio (*t_C_/t_m_*), was investigated. The pull-in voltage versus *N_C_* is shown in Figure 13. This analysis was conducted for *σ_M_* range from 50-100 MPa, *t_C_/t_m_* = 0.16 and *ω* = 30 GHz. It can be seen that a 20 percent reduction in *V_PI_* was achieved with *N_C_* = 24.

**Figure 14.**
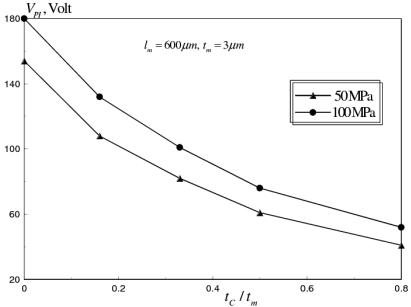
Effect of corrugation thickness ratio on *V_PI_* of corrugated aluminum RF MEMS switch at *σ_M_* range of 50–100 MPa (*l_m_* = 600 *μm, W_m_* = 20 *μm, t_m_* = 3 *μm, N_C_* = 24, *ω* = 30 GHz).

The pull-in voltage versus *t_C_/t_m_* is shown in Figure 14. This analysis was conducted for *σ_M_* range from 50-100 MPa, *N_C_* =24 and *ω* = 30 GHz. It can be seen that proper selection of *t_c_* can dramatically reduce *V_PI_*, up to 60 percent, at the studied range of microfabrication residual stresses.

**Figure 15.**
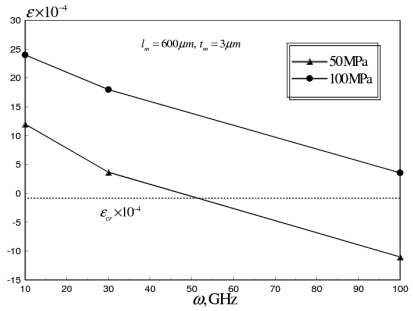
Average axial strain versus *ω* of corrugated aluminum RF MEMS switch at *σ_M_* range of 50-100 MPa (*N_C_* = 24, *t_C_/t_m_* = 0.5).

The average axial strain was also examined for RF switch (*N_C_* = 24, *t_C_/t_m_* = 0.5), at range from 10-100 GHz, as shown in Figure 15. It can be seen that for *σ_M_* = 50 MPa, the buckling failure occurs at *ω* ≈ 50 GHz, while for *σ_M_* = 100 MPa, the power handling capability of the RF switch can be increased beyond 100 GHz.

**Figure 16.**
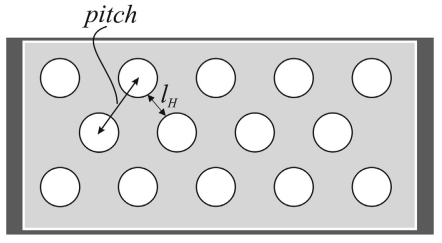
Parameters for the ligament efficiency in RF MEMS switch membrane with through holes.

A very promising design is the addition of holes in to corrugated switch membranes [4,22]. The holes total area can be up to 60 percent of the total surface area of the switch structure [4]. The perforation pattern is mainly characterized by the ligament efficiency, *μ_H_* = *l_H_*/*pitch*, defined as the ratio of the remaining link width to pattern pitch, as seen in Figure 16.

**Figure 17.**
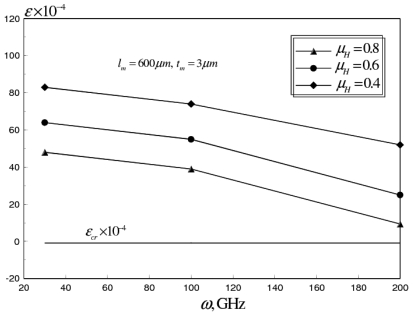
Effect of ligament ratio (*μ_H_*) on average axial strain of corrugated aluminum RF MEMS switch at *ω* range of 30–200 GHz (*σ_M_* = 100 MPa, *N_C_* = 24, *t_C_/t_m_* = 0.5)

The holes allow for the release of part of the residual stresses in the membrane, and reduce the effective Young's modulus of the switch membrane. The reduction of the effective Young's modulus, yields lower values of actuation voltages. Moreover, this reduction increases the positive residual axial strain component, which counteracts the compressive strains generated at higher frequencies due to the skin effect. Therefore, the power handling capability of the RF switch can be increased considerably, without buckling, while still at reliable ranges of actuation voltages. Figure 17 shows the average axial strain versus an operational frequency range 0–200 GHz.

This analysis was conducted for *μ_H_* range from 0.4–0.8, *σ_M_* = 100 MPa, *N_C_* = 24 and *t_C_/t_m_* = 0.5. It can be seen that a higher operational frequency range is achievable for corrugated switch designs with holes (ω > 200 GHz, *μ_H_* = 0.4), compared to *ω* ≈ 100 GHz, for corrugated switch design.

## 6. Conclusions

The commercialization of RF MEMS switches is subject to the elimination of the buckling failure at high RF power and the stiction problem associated with higher actuation voltages. In this paper, a substructured coupled multi-field model was presented to examine the performance of RF MEMS switches at an operational frequency range 0.1–100 GHz. The effect of microfabrication residual stresses and effectiveness of some mechanical approaches has been examined in detail. The coupled model, proposed in this study, represents the first effort to link an actual temperature distribution, due to the skin effect, associated with higher operational frequencies to a coupled structural-electrostatic analysis of RF MEMS switches. Other studies reported in the literature assume a uniform temperature distribution in the switch membrane, which might generate inaccurate predictions of the actual pull-in voltages. The main conclusions from the analyses conducted in this study may be summarized as follows:

1. The temperature rise at higher operational frequencies induces compressive stresses in the switch membrane of flat RF MEMS switches. The induced compressive stresses lead to buckling and device failure for ω ≥ 10 GHz. Moreover, the change in the deformation state of the switch leads to a dramatic increase in the required actuation voltages.
2. In-plane tensile residual stresses generated during microfabrication counteract the induced compressive thermal stresses. In this sense, proper control of microfabrication residual stresses can increase the power carrying capacity of the RF switch. However, higher values of actuation voltages are exhibited because of the membrane stiffening effect.
3. Membrane corrugations, at a distance from the support area can be used to maintain the actuation voltages at lower values. Therefore, using these membranes in conjunction with a proper control of microfabrication residual stresses, increase the reliable range of operation of the RF switch.
4. A better design can be achieved by introducing holes to corrugated switch membranes. These holes help in reducing the effective Young's modulus of the switch membrane. This mainly would yield lower actuation voltages. Moreover, this reduction increases the positive residual axial strain component, which counteracts the compressive strains generated at higher frequencies. Therefore, the power handling capability of the RF switch can be increased considerably, without buckling, while maintaining a suitable actuation voltage. Our analysis of a corrugated switch membrane, with holes at a ligament efficiency of 0.4, shows that this design can increase the reliable operation of the RF switch to ω > 200 GHz.
